# Natural Products: Insights into Leishmaniasis Inflammatory Response

**DOI:** 10.1155/2015/835910

**Published:** 2015-10-11

**Authors:** Igor A. Rodrigues, Ana Maria Mazotto, Verônica Cardoso, Renan L. Alves, Ana Claudia F. Amaral, Jefferson Rocha de Andrade Silva, Anderson S. Pinheiro, Alane B. Vermelho

**Affiliations:** ^1^Departamento de Produtos Naturais e Alimentos, Faculdade de Farmácia, Universidade Federal do Rio de Janeiro, 21941-902 Rio de Janeiro, RJ, Brazil; ^2^Departamento de Microbiologia Geral, Instituto de Microbiologia Paulo de Góes, Universidade Federal do Rio de Janeiro, 21941-902 Rio de Janeiro, RJ, Brazil; ^3^Programa de Pos-Graduação em Imunologia, Instituto de Microbiologia Paulo de Góes, Universidade Federal do Rio de Janeiro, 21941-902 Rio de Janeiro, RJ, Brazil; ^4^Departamento de Produtos Naturais, Farmanguinhos, FIOCRUZ, 21041-250 Rio de Janeiro, RJ, Brazil; ^5^Departamento de Química, Universidade Federal do Amazonas, Japiim, 69077-000 Manaus, AM, Brazil; ^6^Departamento de Bioquímica, Instituto de Química, Universidade Federal do Rio de Janeiro, 21941-909 Rio de Janeiro, RJ, Brazil

## Abstract

Leishmaniasis is a vector-borne disease that affects several populations worldwide, against which there are no vaccines available and the chemotherapy is highly toxic. Depending on the species causing the infection, the disease is characterized by commitment of tissues, including the skin, mucous membranes, and internal organs. Despite the relevance of host inflammatory mediators on parasite burden control, *Leishmania* and host immune cells interaction may generate an exacerbated proinflammatory response that plays an important role in the development of leishmaniasis clinical manifestations. Plant-derived natural products have been recognized as bioactive agents with several properties, including anti-protozoal and anti-inflammatory activities. The present review focuses on the antileishmanial activity of plant-derived natural products that are able to modulate the inflammatory response *in vitro* and *in vivo*. The capability of crude extracts and some isolated substances in promoting an anti-inflammatory response during *Leishmania* infection may be used as part of an effective strategy to fight the disease.

## 1. Introduction

Human leishmaniasis is an infectious disease caused by 20 different* Leishmania* species reported in 98 countries and territories spread across four continents (Africa, Americas, Asia, and Europe). Leishmaniasis is considered a major public health issue as it currently affects 12 million people [1]. The anthroponotic and zoonotic forms of transmission may occur. In the last case, the primary reservoirs of* Leishmania* are sylvatic mammals such as forest rodents, hyraxes, and wild canids. However, urban or domestic dogs are the most relevant species in the epidemiology of this disease [2].

*Leishmania* infection occurs during the hematophagy of female sand flies belonging to* Phlebotomus* (Old World) and* Lutzomyia* (New World) genus. The metacyclic promastigote forms present in the foregut of the sand flies are inoculated in the dermis-epidermis junction of the vertebrate host, infecting cells of the mononuclear phagocyte system [3]. The interaction between parasites and host immune cells leads to an inflammatory response essential for parasite control. However, an exacerbated proinflammatory response may cause tissue damage, such as those easily observed in cutaneous leishmaniasis cases [4, 5]. On the other hand, the lack of an effective inflammatory response may promote increased parasite burden. In this scenario, a moderate inflammatory response would be ideal for an effective control of the disease.

Plants have been long recognized as a rich source of biologically active extracts, essential oils, and isolated substances. In fact, research laboratories around the world search in plants for active substances against diverse illnesses such as microbial and protozoal infections, cancer, diabetes, and inflammatory processes [6]. Indeed, plant-derived natural products such as phenolic compounds, steroids, quinones, coumarins, terpenoids, and alkaloids have been widely investigated for their antileishmanial potential [7, 8].

In the present review, we start with an introduction about the current scenario of leishmaniasis epidemiology and treatment, followed by some highlights on the inflammatory response generated by* Leishmania* infection. The last part of this work focuses on the modulatory effects of plant-derived natural products over inflammatory mediators and their impact on parasite burden* in vivo* and* in vitro*.

## 2. Leishmaniasis: A Global Threat

It is estimated that leishmaniasis has about 1.6 million new cases per year. However, only 600,000 cases are reported annually. Socioeconomic conditions such as poverty and malnutrition, environmental changes such as atmospheric temperature and humidity, ecological conditions affecting the vector, parasite, and its reservoir, and population movements caused by migration and tourism are all risk factors that directly interfere with the world's distribution of leishmaniasis [9–11]. In addition, the ecology of sand fly species also plays a significant role in the spread of the disease [12].

According to geographical criteria, leishmaniasis can be divided into two main syndromes: (1) Old World Leishmaniasis, which includes two clinical manifestations: cutaneous leishmaniasis (CL), a disease confined to the skin, and visceral leishmaniasis (VL), involving the bloodstream and inner organs; (2) New World Leishmaniasis, which includes CL and mucocutaneous leishmaniasis (MCL). The latter involves mucous membranes in addition to the skin. Currently, new terminology regarding leishmaniasis forms was introduced such as mucosal leishmaniasis (ML). ML involves mucosal tissues, particularly those of the upper respiratory tract and oral cavity. It is typically a consequence of infection by New World* Leishmania* species, such as* L. braziliensis, L. panamensis, L. amazonensis*, and* L. guyanensis* [10].

Cutaneous leishmaniasis is found in South America, Asia, Europe, and Africa. Latin America is the most important endemic area, particularly the Amazon. Different* Leishmania* species cause Old World (Eastern hemisphere)* versus* New World (America) CL: in the Old World, the etiologic agents include* L. tropica, L. major*,* L. aethiopica*,* L. infantum*, and* L. donovani; *the main species in the New World are either those of the* L. mexicana* complex (*L. mexicana, L. amazonensis*, and* L. venezuelensis*) or the ones of the subgenus* Viannia* (*L. *(*V.*)* braziliensis, L.* (*V.*)* guyanensis, L. *(*V.*)* panamensis,* and* L.* (*V.*)* peruviana*).

The general term visceral leishmaniasis can refer to different degrees of disease severity, including chronic, subacute, or acute, affecting internal organs, particularly spleen, liver, and bone marrow. The two most important causative agents of VL are* L. donovani*, which shows anthroponotic transmission (human to human), and* L. infantum*, with zoonotic transmission (canine to human). Together, they cause 40,000 deaths* per* year [13].* L. donovani* is only found in the Old World, being responsible for VL cases in East Africa and the northeast of India. On the other hand,* L. infantum* is found in the Mediterranean and in Latin American regions [14]. Over 90% of VL cases occur in Bangladesh, Brazil, Ethiopia, India, South Sudan, and Sudan [11].

## 3. Available Chemotherapy for Leishmaniasis

Chemotherapy is the current method for human leishmaniasis treatment since there are no vaccines available. Usually, the therapeutic approach starts with the use of pentavalent antimonials such as sodium stibogluconate and meglumine antimoniate. However, when these drugs exhibit low efficacy or simply cannot be prescribed for leishmaniasis treatment, second-line drugs are indicated [12, 15, 16].

Several* Leishmania*-killing mechanisms have been attributed to pentavalent antimonials including apoptosis, disturbance of fatty acids *β*-oxidation, adenosine diphosphate phosphorylation, and redox balance. In addition, antimonials inhibit the glycolysis pathway and are able to directly act on infected macrophages eliciting an oxidative/nitrosative stress against internalized parasites [17, 18]. Despite the variety of antileishmanial targets, the use of pentavalent antimonials has been extensively discussed due to their toxic effects to liver and heart tissues. Regarding the use of amphotericin B, this drug targets ergosterol, an essential plasma membrane sterol found in* Leishmania *spp. Also, amphotericin B recognizes cholesterol in mammalian cells, which leads to high toxicity and severe side effects, including kidney failure, anemia, fever, and hypokalemia [19].

Miltefosine and paromomycin are two other drugs that have been introduced for the treatment of leishmaniasis. Miltefosine was the first orally administered drug effective against VL. The mechanism of antileishmanial action of miltefosine remains unclear but apoptosis preceded by drug intracellular accumulation has been described. Other possible mechanisms include cytochrome c oxidase inhibition, which leads to mitochondrial dysfunction and immunomodulation [20]. The recommended dose of miltefosine for VL treatment is approximately 2.5 mg/kg/day for 4 weeks. The long term therapy in conjunction with miltefosine long half-life (about 150 h) can accelerate the onset of drug resistance. Moreover, recent studies have pointed out that miltefosine has a potential teratogenic and abortifacient effect, preventing its prescription during pregnancy [18, 21]. Paromomycin is an aminoglycoside antibiotic that has shown important results in leishmaniasis treatment, mainly for the cutaneous form of the disease [22]. However,* in vitro* studies have already reported the emergence of paromomycin-resistant parasites, compromising its use as a wide antileishmanial agent in the future [23]. In addition, the toxicity of miltefosine and paromomycin has also been described [12].

In summary, the current chemotherapy scenario urges for more efficient and secure antileishmanial treatments, encouraging the search for new bioactive compounds such as those from natural origin. In fact, plant-derived natural products represent a promising class of drug candidates against leishmaniasis.

## 4. Inflammatory Response to* Leishmania* Infection

Parasite-host interaction is a complex process that modulates* Leishmania* infection and the immunological response to it, including inflammation. Several molecules are involved in inflammation during leishmaniasis, such as cytokines and the lipid mediator leukotriene B4 (LTB4). Many of the molecules that promote inflammation also activate phagocytes leading to the production of nitric oxide (NO), the main effector molecule in parasite killing. However, an exacerbated production of these molecules may also lead to tissue damage.

Tumor necrosis factor (TNF) and interleukin-1 (IL-1) are cytokines produced by macrophages after the recognition of pathogens, including* Leishmania*. They promote inflammation by inducing the expression of adhesion molecules (selectin and integrin ligands) on the endothelial surface. TNF- or TNF-receptor 1- (TNFR1-) deficient mice are able to control* L. major* replication but develop larger lesions [24, 25]. The role of IL-1 in leishmaniasis is controversial, as IL-1 contributes to Th1 priming at early infection but worsens the disease outcome in established infection [26].

IL-10 is an important anti-inflammatory cytokine responsible for peripheral tolerance to self-antigens and preventing exacerbated immune responses to foreign antigens. However, when expressed in large quantities, IL-10 may have deleterious effects during leishmaniasis, leading to an early suppression of innate and acquired immune responses, pathogen proliferation, and aggravation of the disease [27]. In leishmaniasis, phagocytes are stimulated to produce IL-10, which leads to a reduced production of cytokines related to the Th1 profile, such as IL-12 and interferon gamma (IFN-*γ*) [28]. This causes a reduction in NO production that consequently reduces the microbicidal capacity of macrophages. IL-10 may be secreted by numerous cells, including macrophages, T cells, and B cells.

The cytokines IL-12 and IL-4 also play an important role during* Leishmania* infection. They define the cell profile through the polarization of CD4+ T cells and modulate the response from other cells [29, 30]. IL-12 activates NK cells and CD8+ T cells, leading to IFN-*γ* production [31]. In addition, IL-12 induces the differentiation of CD4+ T cells to the Th1 profile, which also produces IFN-*γ*, a potent inducer of NO production in macrophages. Thus, IL-12 possesses an indirect microbicidal action. In contrast, IL-4 induces the differentiation of CD4+ T cells to a Th2 profile, which produces IL-4, IL-5, and IL-13. This profile suppresses NO production and leads to an increase in eosinophils [32].

LTB4 is an eicosanoid with chemotactic function synthesized from leukotriene A4 by leukotriene-A4 hydrolase.* In vitro*, LTB4 contributes to the microbicidal action of macrophages through the production of NO and reactive oxygen species while,* in vivo*, LTB4 reduces the parasite load and the footpad swelling [33, 34].

**Figure 1:**
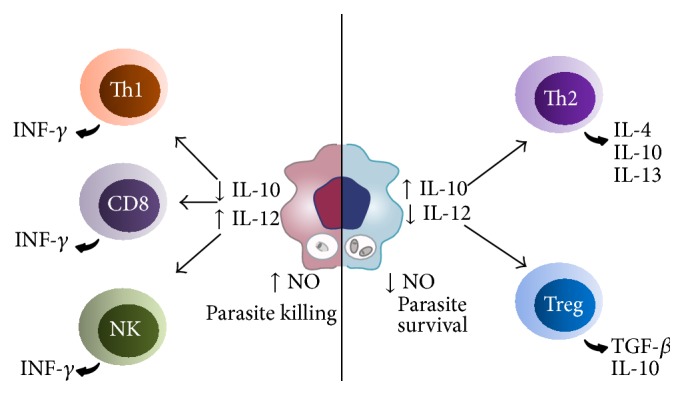
Cytokine profile regulates the type of immune response to* Leishmania* infection. The balance between IL-10 and IL-12 produced by macrophages regulates the parasitic load by controlling NO production, CD4 + T lymphocytes profile, and IFN-y production by NK and CD8 + cells.

The importance of the type of immune response, if Th1 or Th2, lies in the fact that Th1 immune response characterizes the resistance mechanism to* Leishmania* infection, while Th2 response has been associated with susceptibility to parasite infection. The Th1 immune response is associated with production of proinflammatory cytokines such as IFN-*γ*, TNF-*α*, and IL-12, while the susceptibility profile of Th2 response is characterized by anti-inflammatory cytokines expression such as IL-10 and IL-4 (Figure 1) [35].

In humans, protection against VL is mediated by Th1 immune response whereas pathogenesis is associated with Th2 response. Most studies suggest that poor Th1-type responses are associated with severe clinical forms of leishmaniasis [36]. Some studies have demonstrated the importance of proinflammatory cytokines IFN-*γ*, TNF-*α*, and IL-12 in* L. donovani *infection. Depletion of these cytokines aggravated the disease progression or made hosts susceptible to infection by* L. donovani* [37].

However, studies about CL showed that higher frequency of proinflammatory cytokine production leads to larger lesions. Some studies pointed that high production of IFN-*γ*, TNF, and NO is not always beneficial [38]. Thus, inadequately controlled immune responses could potentially lead to pathological manifestations and tissue damage. This is contradictory since many studies pointed out that the Th1-mediated response is important for disease control. The activation of type effector cells that produce the macrophage-activating cytokines (i.e., IFN-*γ*) is necessary for host control over parasite replication [39]. Increasing evidence suggests that the paradigm established about the necessity of a Th1 response for a better prognosis of leishmaniasis is not a rigid concept and the balance between proinflammatory and anti-inflammatory cytokines determines the outcome of the infection [40–42].

## 5. Natural Products Effects on Host Immunological Response

As mentioned earlier, leishmaniasis treatment is primarily based on antimonial compounds followed by amphotericin B as a second choice drug. However, high toxicity, severe side effects, and elevated costs hinder the use of these drugs in countries where leishmaniasis is endemic. In many instances, traditional medicines are the alternative for accessible treatments against parasitic diseases [41]. Unfortunately, most of them are hardly explored and their mechanisms of action are mainly unknown. Plants possess a large repertoire of secondary metabolites that display a wide variety of pharmacological activities. Indeed, numerous plant-derived bioactive compounds have been described, such as terpenoids, flavonoids, alkynes, alkaloids, saponins, sterols, phenylpropanoyl esters, lactones, tannins, and coumarins [43–45].

Traditional herbal medicines are gaining increased attention as they can reduce the risk of chronic diseases and act as antibiotics, antioxidants, and/or immunomodulators. Several studies have described the effects of plant extracts or isolated compounds in immune cells and cytokine production [43]. Thus, the study of active compounds obtained from plants used in traditional medicine plays a pivotal role in the search for new antileishmanial molecules [39, 41].

Several raw extracts from different plants have been shown to exhibit antileishmanial activity, which may not only be due to their direct action on the parasite, but also due to a concomitant effect on the host immune response [41]. Therefore, the search for plant extracts with a wide spectrum of antileishmanial and immunomodulatory activities may enable the discovery of substances suitable for the disease control. Some studies have focused on the effects of leishmanicidal essential oils and plant extracts in the production of pro- and anti-inflammatory soluble mediators. Altogether, these studies suggest that the induction or inhibition of cytokine production is a critical factor for effective parasite destruction without producing excessive tissue damage.  Table 1 summarizes the currently known plant extracts and their effects on inflammatory mediators.

**Figure 2:**
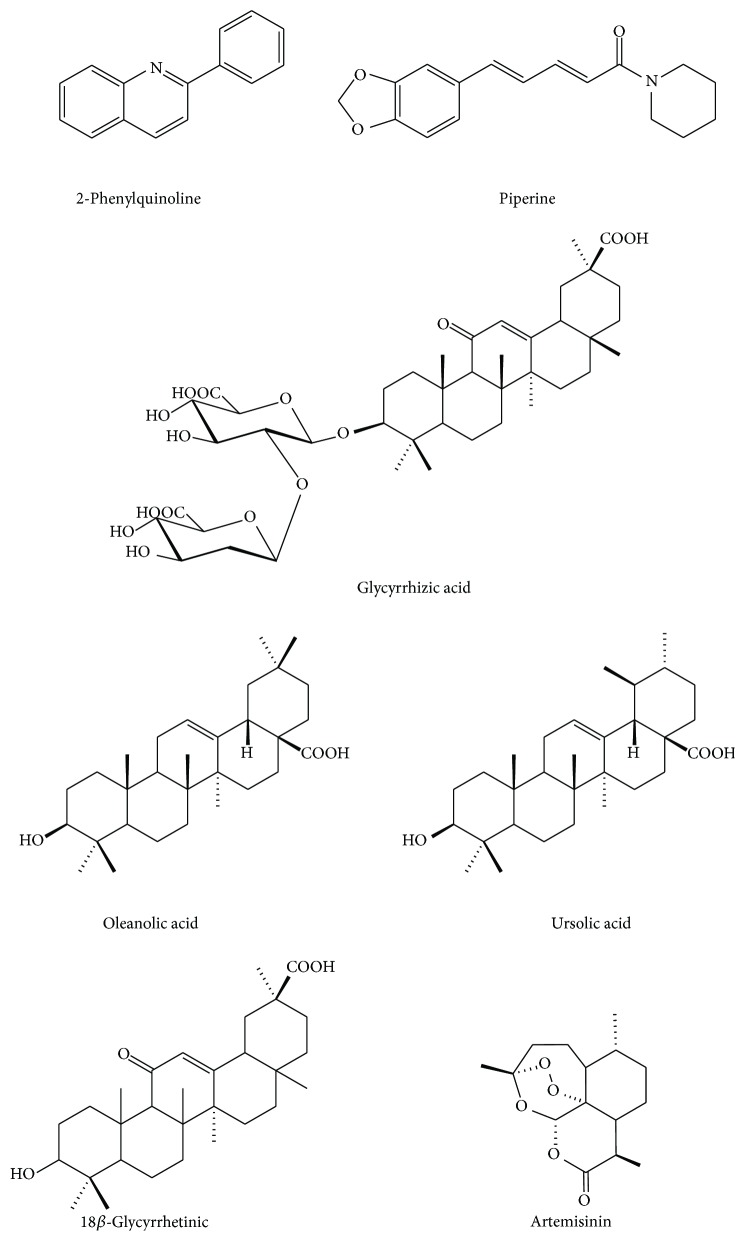
Structures of the compounds assayed* in vitro* and* in vivo* against* Leishmania* species and cytokines activity.

The plant popularly known as Evanta (*Angostura longiflora* (*Krause*)* Kallunki*) is used for the treatment of leishmaniasis and other parasitic diseases in Bolivia [41]. In addition to having direct activity against* L. braziliensis*, Evanta extracts also interfere with the activation of both mouse and human T cells. Calla-Magarinos et al. (2009) [41] showed that the alkaloid-rich extract from Evanta barks (AEE) reduced INF-*γ* expression in J774 and spleen cells, despite its lack of effect on TNF-*α* and NO production. Similar effects were observed in human peripheral blood mononuclear cells (PBMCs). The major compound in the alkaloid-rich extract from Evanta barks is 2-phenylquinoline. Interestingly, the isolated substance (Figure 2) showed a similar effect to that observed for AEE. Moreover, 2-phenylquinoline reduced INF-*γ* production and cell proliferation* in vitro*, suggesting that it may contribute to the control of the chronic inflammatory reaction that characterizes* Leishmania* infection.

Recently, Calla-Magariños et al. (2013) demonstrated that the alkaloid-rich Evanta extract interferes with* in vitro* antigen-specific lymphocyte activation [40]. When spleen cells from* L. braziliensis*-immunized mice were pretreated with AEE and stimulated with* Leishmania* lysate or* Leishmania*-infected bone marrow macrophages (L-BMM), the levels of IFN-*γ* decreased. In addition,* in vivo* treatment with the Evanta extract affected reactivation of primed lymphocytes, reducing the production of IFN-*γ*, IL-12, and TNF-*α* by spleen cells induced with L-BMM. AEE treatment also affected the kinetics of infection. Mice infected with* L. braziliensis* promastigotes in the left hind footpad showed a more effective decrease in the footpad thickness when treated with AEE than those treated with meglumine antimoniate. These results suggest that AEE can control both* Leishmania* infection and the inflammatory reaction against it.

The leaf methanol extract and the essential oil from* Xylopia discreta* display antileishmanial activity and immune stimulatory effects over infected murine macrophages [42]. To evaluate the effects of the methanol extract and the essential oil from* X. discreta*, López et al. (2009) infected J774 cells with* L. panamensis* and measured the levels of proinflammatory mediators. IL-12, IL-10, IL-6, MCP-1, and TNF-*α* were quantified after treatment with different concentrations of* X. discreta* extract or essential oil. No statistical differences in the production of interleukins and TNF-*α* were observed between treated and untreated cells. However, a significant increase in MCP-1 production was observed after cell treatment. Surprisingly, no differences in cytokine production were detected when pentamidine was used as antileishmanial drug [42].

The extract produced from the leaf of Neem (*Azadirachta indica*) presents antileishmanial and immunomodulatory activities [46]. The leaf and seed extracts of* A. indica* were shown to possess immunomodulatory, insecticidal, antiseptic, anticancer, antiviral, antifungal, and antiprotozoal properties. Its oil, bark, and leaf extracts have therapeutic efficacy against leprosy, intestinal helminthiasis, and respiratory disorders in children [47]. Similar to the* X. discreta* extract [42], the ethyl acetate extract fraction of Neem also induces a Th1 response. Cytokine production was evaluated by real time quantitative PCR (RT-qPCR) on THP-1 and PBMCs infected with* L. donovani* strain Dd8. Cells treated with Neem extract showed a significant increase in TNF-*α*, IL-8, and IL-1*β* production, while IL-10 expression was unaltered, indicating a strong Th1 response. However, the expression of TNF-*α* and IFN-*γ* was unaltered in spleen tissue (*in vivo* analysis), whereas the expression of Th2 cytokines (IL-10, IL-4, and TGF-*β*) was significantly reduced [46]. These results suggest that the leaf extract of Neem induces a protective immune polarization during leishmaniasis.

Chouhan et al. (2015) evaluated the antileishmanial and immunomodulatory activities of the ethanol extract of leaves (ALE), seeds (ASE), and bark (ABH) from* A. indica*. In contrast to Dayakar et al. (2015) [46], they used other parts of the plant and different extraction methods. ABH is not effective against* L. donovani* promastigotes, while ALE and ASE exhibited leishmanicidal activity in both promastigote and amastigote cells. Sera of treated mice infected with* L. donovani* were analyzed for IgG2a (induced by INF-*γ*) and IgG1 (induced by IL-4) levels. Highest levels of IgG2a are indicative of Th1 response, while IgG1 indicate Th2 activation. ALE and ASE stimulated the production of high levels of IgG2a and low levels of IgG1. As expected, ALE and ASE treatment induced NO generation by macrophages primed with SLA. Confirming these results, Th1/Th2 cytokine levels were quantified in culture supernatants of spleen cells from animals treated with ALE and ASE. The extracts significantly increased the levels of Th1 cytokines, such as INF-*γ*, TNF-*α*, and IL-2, and decreased the IL-10 and IL-4 levels [47]. Although Chouhan et al. (2015) and Dayakar et al. (2015) have used different parts of* A. indica*, both of them showed the proinflammatory effects of bioactive molecules derived from this plant.

The genus* Laennecia* and the correlated genus* Conyza* are known to produce bioactive substances displaying antimicrobial, antiparasitic, antidiarrhoeal, antinociceptive, antioxidant, and anti-inflammatory activities. Aiming to evaluate the potential of* L. confusa*, Ruiz et al. (2012) investigated the inhibitory effect of different extracts from its stems against several pathogenic microorganisms. In addition, the anti-inflammatory activity of these extracts was evaluated. The aqueous and chloroform extracts, as well as a chloroform fraction, named, CE2, presented antiparasitic activity against* L. donovani*. However, these extracts and fractions did not affect the production of proinflammatory cytokines (IL-6) in THP-1 cells [48].

A similar approach was conducted by Bolivar et al. (2011) with* Galium mexicanum* and by Paredes et al. (2013) with* Lopezia racemosa*, with both of them being traditional medicinal plants used in Mexico [49, 50]. Flavonoids, iridoid glycosides, iridoid acids, triterpene saponins, and anthraquinones have been isolated from the* Galium* genus. Among the* G. mexicanum* extracts and fractions analyzed, the hexane fractions HE 5 and HE 14b presented anti-*L. donovani* promastigotes activity, while the hexane fraction HE 5 and methanol fractions ME 13–15 reduced the LPS-induced macrophage production of IL-6, suggesting an anti-inflammatory character of these samples [49].

The aerial parts of* L. racemosa* were submitted to extraction with various solvents and the extracts were fractionated. The hexane fractions HF 11–14b, methanol fractions MF 28–36, and the chloroform extract were able to inhibit* L. donovani* growth. In relation to the reduction of IL-6 production by macrophages exposed to LPS, the fractions HF 11–14b showed significant anti-inflammatory activity by reducing the secretion of the aforementioned cytokine [50].

*Croton caudatus* leaves extract is a promising extract against visceral leishmaniasis. Stems and leaves of* C. caudatus* have been used for the treatment of rheumatic arthritis, malaria, convulsions, ardent fever, numbness, worm-infested animals, vomiting, and dysentery in India [39]. Terpenes as crotocaudin, isocrotocaudin, crotoncaudatin, and crocaudatol have been isolated from this extract. Dey et al. (2015) demonstrated that the semipurified hexane extract of* C. caudatus* leaves (JDHex) inhibited the proliferation of* L. donovani* promastigotes (IC_50_ = 10 *μ*g/mL) and intracellular amastigotes (IC_50_ = 2.5 *μ*g/mL). To evaluate the immunomodulatory activity of JDHex, the production of proinflammatory cytokines, such as IL-12 and TNF-*α*, as well as anti-inflammatory cytokines, IL-10 and TGF-*β*, was investigated* in vitro* and* in vivo*.* L. donovani*-infected murine peritoneal macrophages treated with JDHex showed an increase in intracellular IL-12 (p70 fraction) and a reduction in TGF-*β* and IL-10 production. In addition, JDHex induced an increase in NO that could be directly correlated with the induction of TNF-*α* expression in infected macrophages. These results suggest that the immunomodulatory activity of JDHex occurs via a Th1 response.* In vivo* experiments performed with mice infected with* L. donovani* and treated orally with different concentrations of JDHex for 5 days after 1 month of infection showed that treated mice had an induction in IFN-*γ* production. In addition, the parasite load in spleen was reduced dose-dependently. As JDHex was efficient against* L. donovani* intracellular amastigotes, the authors suggested that the proinflammatory activity of JDHex may be useful for antileishmanial therapy [39]. Using a similar* in vivo* model, Bhattacharjee et al. (2012) and Chouhan et al. (2015) found comparable results for treatment of* L. donovani* with glycyrrhizic acid (Figure 1) extract from liquorice (*Glycyrrhiza glabra*) and ethanol extract of* A. indica*, respectively [37, 47].

In accordance with Dey et al. (2015) [39], Yamamoto et al. (2014) also described an antileishmanial compound that induces Th1 response.* L. amazonensis*-infected mice were treated with a triterpene-rich fraction of* Bacchari suncinella* during five days. The analysis of immune response revealed that treated mice presented higher levels of IL-12 and IFN-*γ* than the control group. Treatment with the triterpenic fraction reduced the size of lesions, as well as the parasitism and the parasite load [51]. It is worth noting that the triterpenic fraction of* B. suncinella* stimulated the inflammatory process while reducing the size of mice lesions.

The flavonoid-rich* Artemisia annua* L. extract has been shown to possess antioxidant, antimicrobial, and anti-inflammatory activities [52]. Studies carried out with the leaves and seeds of* A. annua* against* L. donovani*-infected mice caused increased production of Th1 cytokines (IFN-*γ*) and a simultaneous decrease in Th2 cytokines (IL-4 and IL-10). Moreover,* A. annua* extracts resulted in higher CD4+ and CD8+ T cell numbers, lymphoproliferation, upregulation of costimulatory molecules (CD80 and CD86) on APCs, and generation of NO [53].

The nor-triterpene 6*α*,7*α*,15*β*,16*β*,24-pentacetoxy-22*α*-carbometoxy-21*β*,22*β*-epoxy-18*β*-hydroxy-27,30-bisnor-3,4-secofriedela-1,20(29)-dien-3,4 R-olide (LLD-3), extracted from* Lophanthera lactescens* Ducke, showed a remarkable antileishmanial activity against intracellular amastigotes (IC_50_ = 0.41 *μ*g/mL) but no cytotoxicity to mouse peritoneal macrophages or B cells, which makes it a promising drug candidate for leishmaniasis treatment [54]. In addition, piperine (Figure 1), the main alkaloid of* Piper nigrum*, and its analogue phenylamide are active against* L. amazonensis* promastigotes and amastigotes. They act synergistically to boost the leishmanicidal effect and reduce the NO production in infected macrophages [55]. The hexane extract of the twigs of* Nectandra leucantha* Nees and Mart displayed activity against the promastigote forms of* L. donovani*. Isolated phenylpropanoid dimers suppressed the production of disease exacerbatory cytokines IL-6 and IL-10 but had minimal effect on NO production in* L. donovani*-infected macrophages. Thus, the antileishmanial activities appear to be mediated by molecular mechanisms that are independent of NO production [56].

## 6. Conclusion

Promising drug candidates for leishmaniasis treatment should be able to eliminate the parasite but also elicit an appropriate immune response. Plant-derived natural products such as crude extracts, purified fractions, or isolated substances have demonstrated their effectiveness as immunomodulatory agents. The anti-inflammatory activity of the natural products pointed here could be useful for the control of an exacerbated proinflammatory response, ameliorating leishmaniasis clinical symptoms, such as tissue damage.

## Figures and Tables

**Table 1:** Plant extracts and isolated compounds with antileishmanial and immunomodulatory activities.

Plant species	Substance or extract	*In vitro* activity (IC_50_)	*In vivo* in mice	Substance cytokines activity	Reference
*Glycyrrhiza glabra* L.	18*β*-glycyrrhetinic acid	*L*. *donovani* 4.6 *μ*g/mL	*L*. *donovani* 50 mg/kg/day	Reduces levels of IL-10 and IL-4, but increases levels of IL-12, IFN-*γ*, TNF-*α*, and inducible NO synthase	[57]

*Tanacetum parthenium *	Parthenolide	*L*. *amazonensis* 0.37 *μ*g/mL	—	Inhibits IB kinase *β*	[58]

*Baccharis uncinella *	Oleanolic acid and ursolic acid	—	*L*. *amazonensis* 1 and 5 mg/kg/day	Increases IL-12 and IFN-*γ* cytokines	[51, 59]

*Dictyota pfaffii *	Dolabelladienetriol	*L*. *amazonensis* 43.9 *μ*M	—	Diminishes TNF-*α* and TGF-*β* production in uninfected and *Leishmania*-infected macrophages	[60]

*Artemisia indica *	Artemisinin	*L*. *donovani*, *L*. *infantum*, *L*. *tropica*, *L*. *braziliensis*, *L*. *mexicana,* and *L*. *amazonensis* 100 *μ*M to 120 *μ*M	*L*. *donovani* 10 mg/kg and 25 mg/kg body weight	Restores Th1 cytokines (interferon-gamma and interleukin-2)	[61]

*Glycyrrhiza glabra *	Glycyrrhizic acid	—	*L*. *donovani* 1, 10, 25, 50, 75, or 100 mg/kg body weight/day	Enhances the expression of IL-12 and TNF-*α*, in parallel with a downregulation of IL-10 and TGF-*β*	[37]

*Nectandra leucantha *	(a) Dehydrodieugenol B, (b) 1-(8-propenyl)-3-[3′-methoxy-1′-(8-propenyl)phenoxy]-4,5-dimethoxybenzene, and (c) 1-(7R-hydroxy-8-propenyl)-3-[3′-methoxy-1′-(8′-propenyl)-phenoxy]-4-hydroxy-5-methoxybenzene	(a) 26.7 *μ*M (*L*. *donovani*), (b) 17.8 *μ*M (*L*. *donovani*), and (c) 101.9 *μ*M (*L*. *donovani*)	—	((a) to (c)) Reduced production of IL-6 and IL-10. Minimal effect on nitric oxide production in *L*. *donovani*-infected macrophages	[56]

*Quassia amara *	Quassin	—	—	Upregulating proinflammatory cytokines such as TNF-*α* and IL-12	[62]

*Vitis vinifera *	Resveratrol	*L*. *amazonensis* Antipromastigote activity (27 ± 0.59 *μ*M) Antiamastigote activity (42 ± 7.18 *μ*M)	—	Decreases the levels of the proinflammatory cytokine TNF-*α* in infected macrophages stimulated with IFN-*γ*	[63]

*Raputia heptaphylla *	11*α*,19*β*-dihydroxy-7-acetoxy-7- deoxoichangin	*L*. (V) *panamensis* J774.2 EC_50_ = 7.9 *μ*M; hDCs EC_50_ = 25.5 *μ*M	—	Increases on the production of IL-12p70, TNF-*α*, and NO, as also, in the number of hDCs HLA-DR-positive in treated infected hDCs	[64]

*Galipea longiflora *	Crude extract containing 13 different quinolinic alkaloids and 2-phenylquinoline as major compounds	*L*. *braziliensis* IC_90_ = 20 *μ*g/mL	*L*. *braziliensis* 6.25 and 12.5 mg/kg/day	Reduced production of IFN-*γ*, IL-12, and TNF-*α* by spleen cells. Reduced the inflammatory reaction in mice infected with *L*. *braziliensis* promastigotes	[40, 41]

*Xylopia discreta *	(a) Methanol extracts containing ~50% of alkaloids and terpenes (*β*-pyrenes, camphene, *β*-myrcene, and 1,8 cineol) and (b) essential oil	(a) *L*. *panamensis* in J774 LC_50_ = 598.37 *μ*g/mL and EC_50_ = 9.32 *μ*g/mL, (b) *L*. *panamensis* in J774 LC_50_ = 857.7 *μ*g/mL and EC_50_ = 37.5 *μ*g/mL, (a) *L*. *panamensis* in U937 LC50 = 698,45 *μ*g/mL and EC50 = 6,35 *μ*g/mL, (b) *L*. *panamensis* in U937 LC50 = 160 *μ*g/mL and EC50 = 6.25 *μ*g/mL	—	Increased the secretion of MCP-1 by U937 and J774 cell lines	[42]

*Galium mexicanum *	Hexane fraction (HE 5)	*L*. *donovani* MIC = 333 *μ*g/mL	—	Reduced production of IL-6 in THP-1 cells	[49]

*Laennecia confusa *	(a) Aqueous extract and chloroform extracts (antileishmanicidal), (b) methanol, and (c) chloroform extracts (anti-inflammatory)	*L*. *donovani* (a) IC_50_ = 20 *μ*g/mL and IC_50_ = 20 *μ*g/mL	—	(b) Reduced IL-6 production in THP-1 cells	[48]

*Azadirachta indica *	Ethyl acetate extract fraction	*L*. *donovani* IC_50_ = 52.4 *μ*g/mL	*L*. *donovani* 100 mg/kg body weight	Increased the expression of TNF-*α*, IL-8, and IL-1*β* in THP-1 cells and TNF-*α*, IFN-*γ* in PBMCs	[46]

*Azadirachta indica *	Leaves ethanol extract (ALE) and seeds ethanol extract (ASE)	Antipromastigote IC_50_ = 34 and 77,66 *μ*g/mL (ALE and ASE). Antiamastigote IC_50_ = 17,66 and 24,66 *μ*g/mL (ALE and ASE)	—	Increased the expression of INF-*γ*, TNF-*α*, and IL-2 and declined in IL-4 and IL-10 levels in spleen cells	[47]

*Lopezia racemosa *	Hexane extract fractions (HE11–14b)	IC_50_ = 30,66 *μ*g/mL	—	Reduced IL-6 production in THP-1 cells	[50]

*Croton caudatus *	Semipurified hexane extract (JDHex)	*L*. *donovani* Antipromastigote IC_50_ = 10 *μ*g/mL. Antiamastigote IC_50_ = 2.5 *μ*g/mL	*L*. *donovani* 1.25, 2.5, 3.75 or 5 mg/kg body weight for five days	Increased the production of IL-12 and TNF-*α* in murine peritoneal macrophages *in vitro*. Increased the intracellular IFN-*γ* and decreased the IL-10 production in CD4^+^ T cells *in vivo *	[39]

*Baccharis uncinella *	Triterpenic purified fraction containing oleanolic and ursolic acids	—	*L*. *amazonensis* 1.0 mg/kg and 5.0 mg/kg for five days	Increased the IL-12 and IFN-*γ* production in mice	[51]

*Sambucus nigra *	Commercial preparation (Sambucol)	—	*L*. *major* 25 *μ*L twice a day	Increased the production of IL-1*β*, IL-6, IL-8, and TNF-*α* by human monocytes	[65]

*Syzygium cumini *	Essential oil (ScEO) containing as major component *α*-pinene	*L*. *amazonensis* Antipromastigote IC_50_ = 19.7 *μ*g/mL	—	Increased in lysosomal volume, phagocytosis, and NO production by peritoneal macrophages	[66]

*Artemisia annua *	Hexane extract	—	*L*. *donovani* 50, 100 and 200 mg/kg body weight daily for ten days	Increased in levels of IFN-*γ* and reduction of levels of IL-4 and IL-10 in serum and culture supernatant of lymphocytes from mice	[53]

*Piper nigrum *	Alkaloids (piperine and analogue phenylamide)	*L*. *amazonensis* Antipromastigote IC_50_ = 14.2 *μ*M. Antiamastigote IC_50_ = 28 *μ*M	—	Suppressed MCP-1, TNF-*α*, NF-KB activation, and NO production *in vitro* and *in vivo* and showed anti-inflammatory properties	[55, 67]

*Tinospora cordifolia *	Pure herb extract (tablet form)	—	*Leishmania donovani* 100 mg/kg b.wt. for 15 days daily	Enhanced proliferation and differentiation of lymphocytes and induced Th1 immune response and IFN-*γ* and IL-2, but declined IL-4 and IL-2 levels	[68]

*Withania somnifera *	Aqueous extract	—	*L*. *donovani* 5 mg/kg/day	Increased antileishmanial efficacy of cisplatin. Increased in the levels of IFN-*γ* and IL-2 (Th1-type immunity) and the levels of IgG2 over IgG1. Decreased in levels of IL-4 and IL-10	[69]

*Lophanthera lactescens *	6*α*,7*α*,15*β*,16*β*,24-pentacetoxy-22*α*-carbomethoxy-21*β*, 22*β*-epoxy-18*β*−hydroxy-27,30-bisnor-3,4-secofriedela-1,20 (29)-dien-3,4 R-olide (LLD-3)	*L*. *amazonensis* . Antiamastigote IC_50_ = 0.41 *μ*g/mL	—	Affected proliferation of naïve or activated B and T cells, as well as the B cells immunoglobulin synthesis	[54]
